# Immune microenvironment dynamics in breast cancer during pregnancy: impact of gestational age on tumor-infiltrating lymphocytes and prognosis

**DOI:** 10.3389/fonc.2023.1116569

**Published:** 2023-08-21

**Authors:** Elham Sajjadi, Konstantinos Venetis, Mariia Ivanova, Marianna Noale, Concetta Blundo, Eugenia Di Loreto, Giovanna Scarfone, Stefano Ferrero, Stefania Maggi, Paolo Veronesi, Viviana E. Galimberti, Giuseppe Viale, Fedro A. Peccatori, Nicola Fusco, Elena Guerini-Rocco

**Affiliations:** ^1^ Department of Oncology and Hemato-Oncology, University of Milan, Milan, Italy; ^2^ Division of Pathology, IEO, European Institute of Oncology IRCCS, Milan, Italy; ^3^ Neuroscience Institute Aging Branch, National Research Council (CNR), Padua, Italy; ^4^ Breast Surgery Unit, Fondazione IRCCS Ca’ Granda—Ospedale Maggiore Policlinico, Milan, Italy; ^5^ Gynecology Unit, Fondazione IRCCS Ca’ Granda—Ospedale Maggiore Policlinico, Milan, Italy; ^6^ Division of Pathology, Fondazione IRCCS Ca’ Granda—Ospedale Maggiore Policlinico, Milan, Italy; ^7^ Department of Biomedical, Surgical, and Dental Sciences, University of Milan, Milan, Italy; ^8^ Division of Breast Surgery, IEO, European Institute of Oncology IRCCS, Milan, Italy; ^9^ Fertility and Procreation Unit, Division of Gynecologic Oncology, IEO European Institute of Oncology IRCCS, Milan, Italy

**Keywords:** breast cancer during pregnancy, pregnancy-associated breast cancer, PD-L1, Foxp3, tumor microenvironment, breast cancer, tumor-infiltrating lymphocytes (TILs)

## Abstract

**Background:**

Breast cancer during pregnancy (PrBC) is a rare condition known for its aggressive clinical behavior. The presence of tumor-infiltrating lymphocytes (TILs) has been shown to have a significant impact on the prognosis of these patients. Despite some biological characteristics of the tumor that may differ depending on the gestational age, little is known about the dynamics of the immune landscape within the tumor microenvironment (TME) in PrBC. Therefore, in this study, our objective was to gain comprehensive insights into the relationship between gestational age at breast cancer diagnosis and the composition of the TME.

**Methods:**

*n* = 108 PrBC were selected from our institutional registry and categorized based on the gestational age by trimester. For all cases, TILs were profiled according to the International TILs Working Group recommendations, and subtyped by CD4, CD8, and forkhead box P3 (FOXP3) immunohistochemistry. PD-L1 was tested according to the combined positive score (CPS) using the IHC 22C3 pharmDx assay, with a cutoff value of ≥10 for positivity. The statistical approach encompassed Fisher’s and Chi-squared tests, with appropriate adjustments for multiple comparisons, logistic regression models, and survival analyses based on the Kaplan–Meier method.

**Results:**

The proportion of patients with poorly differentiated (G3) neoplasms increased as the gestational age advanced (first trimester, *n* = 25, 56.8%; second trimester, *n* = 27, 69.2%; third trimester, *n* = 21, 87.5%; *p* = 0.03). The histologic subtypes as well as the hormone receptor (HR) and HER2 status did not show significant changes across different pregnancy trimesters. In the HR+/HER2– subtype, there was a higher proportion of tumors with high/moderate TILs in the early phases of pregnancy, similar to FOXP3 expression (TILs: first trimester, *n* = 10, 35.7%; second trimester, *n* = 2, 10.5%; third trimester, *n* = 0; *p* = 0.02; FOXP3: first trimester, *n* = 10, 40%; second trimester, *n* = 3, 15.8%; third trimester, *n* = 0; *p* = 0.03). The median follow-up for our cohort was 81 months. Patients who relapsed after a breast cancer diagnosis during the first trimester were more frequently PD-L1-negative, unlike those with no disease recurrence (*n* = 9, 100% vs. *n* = 9, 56.3%; *p* = 0.03; hormone therapy and *n* = 9, 100% vs. *n* = 7, 53.9%; *p* = 0.02; chemotherapy). No statistically significant differences were seen among the three trimesters in terms of survival outcome.

**Conclusion:**

The TME dynamics of HR+/HER2− PrBC vary based on gestational age, suggesting that immune tolerance expression during later gestational age could explain the increased aggressiveness of tumors diagnosed at that stage.

## 1 Introduction

Breast cancer is a commonly occurring malignancy during pregnancy, accounting, albeit rare, for ~4% of early-onset breast cancers (EOBCs) (1, 2). The clinical characteristics of breast cancer during pregnancy (PrBC) often manifest as advanced tumor stage, nodal involvement, and poorly differentiated histologies, indicating a more aggressive disease presentation (3, 4). Existing guidelines recommend the adoption of breast cancer standard treatment protocols for patients with PrBC (5, 6). However, it is important to consider potential modifications to these approaches due to possible delays in the diagnosis caused by physiological changes that occur during pregnancy (6–8).

There have been reports highlighting the similarities between the immunological characteristics and mechanisms at the maternal–fetal interface and those observed in tumors (9, 10). These similarities include the mechanisms involved in maternal–fetal tolerance and tumor-host immunoediting (11). Regulatory T cells (Tregs) and programmed death-ligand 1 (PD-L1) play pivotal roles in embryo implantation and induction of maternal–fetal tolerance during pregnancy (12–14). However, the potential impact of these changes on breast cancer development and progression remains a subject of debate (15). In our previous work, we conducted a comprehensive characterization of the tumor microenvironment (TME) in a large cohort of PrBC cases (16). Our findings revealed distinctive immunological and biological features of PrBC compared to conventional EOBC. Significant differences were observed between the two groups regarding the presence of tumor-infiltrating lymphocytes (TILs) and their subpopulations, as well as the expression of PD-L1. Patients with PrBC exhibited a significantly higher risk of relapse and mortality compared to those with EOBC, particularly among those with CD8+ TILs. However, the prevalence of TILs and their prognostic significance in PrBC remain controversial, with some studies reporting a low prevalence of TILs and others observing similar clinical outcomes to EOBC (17–24).

Several studies have examined the clinicopathological alterations and prognoses associated with breast cancer diagnosed at different gestational ages (3, 25). It has been observed that the histopathological characteristics of the tumors vary significantly across gestational trimesters. Specifically, individuals diagnosed later during pregnancy often exhibit a hormone receptor (HR)-negative phenotype and experience worse clinical outcomes (3). These findings suggest the existence of distinct biological profiles of PrBC that are influenced by gestational age. Taken together, there is a need to elucidate PrBC biological dynamics and better understand the role of the immune system in these tumors. Our objective was to offer a comprehensive understanding of the correlation between the gestational age at PrBC and the varying composition of the TME.

## 2 Materials and methods

### 2.1 Patients and tissue specimens

The patients included in this study were jointly diagnosed and treated at the European Institute of Oncology (IEO), Milan, Italy, and Fondazione IRCCS Ca` Granda – Ospedale Maggiore Policlinico, Milan, Italy, between February 2002 and November 2017. The study received ethical approval from the local Ethical Committees under protocol numbers #620_2018bis and #UID3472. From our datasets, we retrieved all patients with PrBC and categorized them based on the trimester in which they were diagnosed. Representative formalin-fixed, paraffin-embedded (FFPE) blocks were carefully selected to construct tissue microarrays (TMAs) for subsequent analyses. Specifically, we generated four TMAs, each containing 180 tumor cores, resulting in a total of 720 tissue spots (with an average of 6.9 tumor samples per patient; range, 5–7 samples). For each case, the TMA sampling included both the core and periphery (i.e., invasive front) of the tumor, as well as matched normal epithelial breast tissue (i.e., glandular tissue with at least one non-neoplastic terminal ductal-lobular unit adjacent to the tumor). Our TMA protocol was optimized for immunohistochemistry (IHC) studies targeting intratumor heterogeneity in FFPE archival tissue blocks of breast cancers (26). Each case underwent thorough review, reclassification, and regrading based on the latest World Health Organization (WHO) classification of breast tumors (27) and the Nottingham histologic grading system (28). Pathologic restaging was performed following the 8th edition of the American Joint Committee on Cancer (AJCC) Cancer Staging Manual (29). The molecular subtypes of breast cancer were determined based on the status of ER, PgR, Ki67, and HER2, following the recommendations of the St. Gallen International Expert Consensus (30).

### 2.2 Tumor-infiltrating lymphocyte analysis

TIL levels were assessed on 4-µm-thick hematoxylin and eosin-stained full-face sections at a ×200 magnification based on the recommendations of the International TILs Working Group (31). TIL percentage was reported only for the stromal compartment as the area of stromal tissue occupied by mononuclear inflammatory cells (including lymphocytes and plasma cells) over the total intratumoral stromal area. TILs outside of the tumor border and around ductal carcinoma *in situ* and normal terminal duct-lobular units were not counted. TIL percentage was recorded both as a continuous value and as sub-categories [i.e., negative (<1%), low (1%–20%), moderate (21%–50%), and high (>50%)].

### 2.3 Immunohistochemical analysis

The HR [i.e., estrogen receptor (ER) and progesterone receptor (PgR)], Ki67, and HER2 status were updated according to the latest breast biomarker reporting guidelines v1.5.0.1 published by the College of American Pathologists in March 2023 (available at: https://www.cap.org/protocols-and-guidelines/cancer-reporting-tools/cancer-protocol-templates, accessed 20 May 2023). HER2-low and ER-low tumors were identified using the established methodologies comprehensively described in previous studies (32–34). Subsequently, lymphocyte subtyping was performed by IHC using antibodies against CD4, CD8, and forkhead box P3 (FOXP3) on a Dako Omnis automated staining platform (Agilent, Santa Clara, CA, USA), as previously described (35–37). The presence and relative proportions of CD4−, CD8−, and FOXP3+ cells within the TME were evaluated as the percentage of positive TILs (31, 38). Then, CD4 and FOXP3 were recorded as dichotomous variables based on the cutoff value of 1%. CD8 was categorized as negative (<1%), low (1%–30%), moderate (31%–50%), and high (>50%). PD-L1 was tested according to the combined positive score (CPS) using the IHC 22C3 pharmDx assay on a Dako Link 48 platform (Agilent, Santa Clara, CA, USA), with a cutoff value of ≥10 for positivity (39–41). For each run, both positive and negative controls were included. Necrotic areas, as well as intraductal components, were excluded from the analysis. The methods and scoring systems employed are detailed in **Supplementary Table S1**.

### 2.4 Statistical analysis

Categorical variables were summarized as counts and percentages, while for continuous variables, means and standard deviations (SD) or median and Quartile 1 (Q1), Quartile 3 (Q3) were used. Normal distributions of continuous variables were tested using the Shapiro–Wilk test. Differences in the baseline characteristics between trimesters were assessed using Fisher’s exact or Chi-squared tests, and Wilcoxon rank-sum test or generalized linear models after testing for homoscedasticity (Levene test), for categorical and continuous variables, respectively. Likewise, the differences between patients who experienced progression and patients who did not, and between patients who died during follow-up and patients alive at the end of the follow-up were analyzed. The association with cancer progression or death during the follow-up was analyzed by survival analysis according to the Kaplan–Meier method and the log-rank test. Two-tailed *p*-values <0.05 were considered statistically significant. The analyses were performed using SAS statistical package, version 9.4 (SAS Institute Inc., Cary, NC).

## 3 Results

### 3.1 Correlation between gestational age and PrBC clinicopathological features

**Figure 1:**
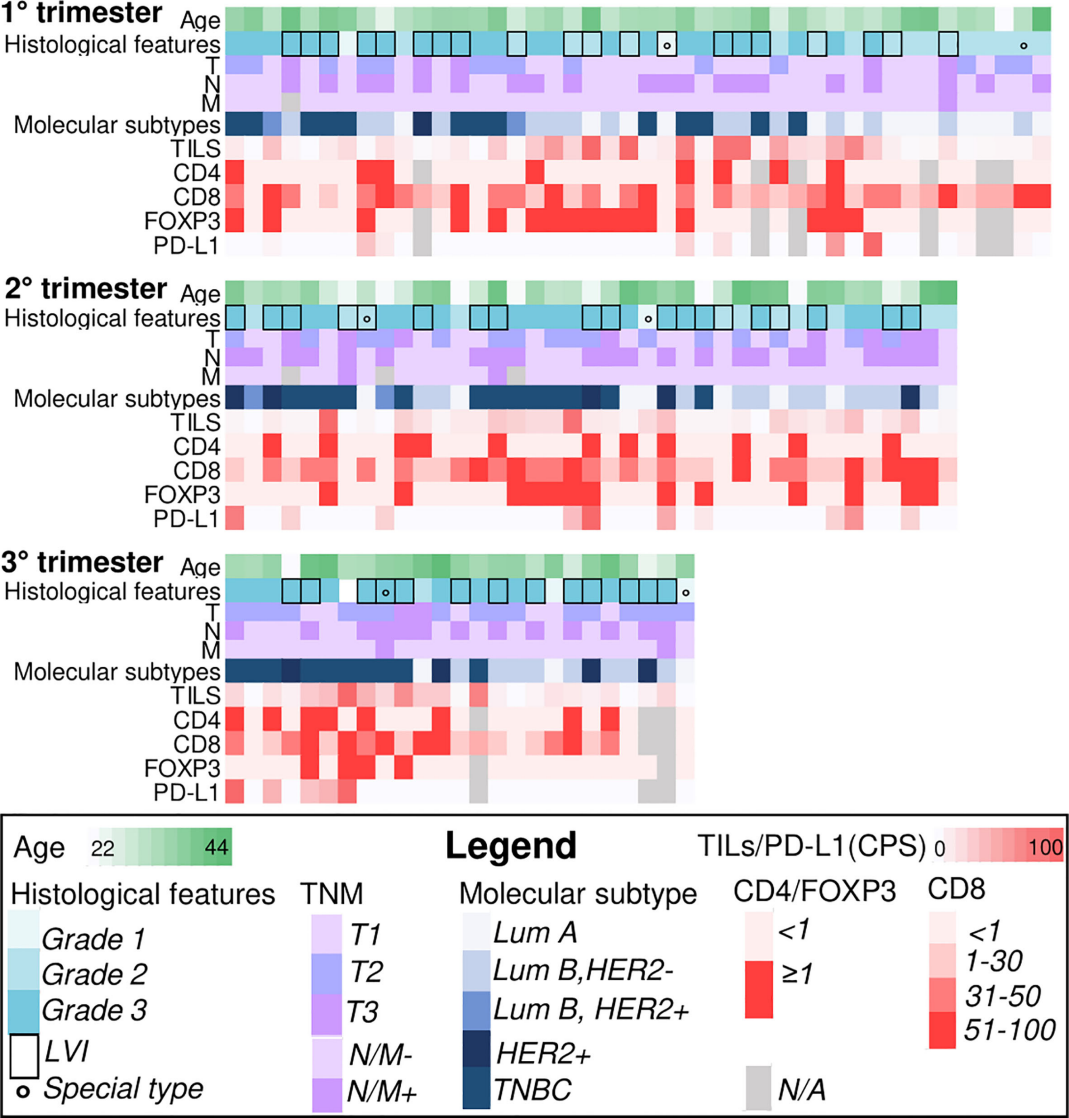
Heatmaps illustrating selected clinicopathologic and immune-related features of breast cancers during pregnancy (PrBC) categorized by trimester. Each column represents a patient, and each row represents a specific parameter, color-coded according to the legend below. TILs, tumor-infiltrating lymphocytes; FOXP3, forkhead box P3; PD-L1, programmed death-ligand 1; LVI, lymph-vascular invasion; LumA, luminal A; LumB, luminal B; TNBC, triple-negative breast cancer; N/A, not available.

A total of 108 women with PrBC were included in this study (age range, 22–44 years; follow-up time, 1–247 months; median time, 81 months). The majority of the patients were diagnosed during the first trimester (*n* = 44, 40.7%) followed by the second trimester (*n* = 39, 36.1%) and the third trimester (*n* = 25, 23.2%). The demographic and clinicopathologic characteristics of patients in each trimester of pregnancy are provided in **Table 1**, while the heatmap in **Figure 1** presents a detailed individual-level analysis of these characteristics. The proportion of patients with high histologic grades (G3) demonstrated a significant increase with advancing gestational age (first trimester: *n* = 25, 56.8%; second trimester: *n* = 27, 69.2%; third trimester: *n* = 21, 87.5%; *p* = 0.03). This observation was accompanied by a lower proportion of patients with low tumor stage (T1) in the later periods (first trimester: *n* = 25, 56.8%; second trimester: *n* = 15, 38.5%; third trimester: *n* = 6, 24.0%; *p* = 0.02), suggesting a more aggressive tumor behavior associated with increased gestational age. However, there were no significant changes in breast cancer subtypes or the HR and HER2 status according to the trimester of pregnancy (**Table 1**). Additionally, no statistically significant associations were found when comparing the prevalence of high histologic grade (G3) with breast cancer subtypes in patients diagnosed with PrBC in the last trimester (**Supplementary Table S2**).

**Table 1:** Clinicopathological characteristics of the patients included in the study, categorized according to the respective pregnancy trimester.

	First trimester *n* = 44	Second trimester *n* = 39	Third trimester *n* = 25	*p-value*
Age at diagnosis, year				0.139
Mean ± SD	34.5 ± 4.7	35.2 ± 3.7	36.6 ± 3.9	
Min, max	22, 44	29, 41	27, 43	
Histological type, *n* (%)				0.759
NST (ductal)	42 (95.5)	37 (94.5)	23 (92.0)	
Other	2 (4.5)	2 (5.1)		
LVI, *n* (%)	20 (45.5)	19 (48.7)	13 (52.0)	0.869
T, *n* (%)				
T1	25 (56.8)	15 (38.5)	6 (24.0)	
T2	14 (31.8)	16 (41.0)	17 (68.0)	
T3/4	5 (11.4)	8 (20.5)	2 (8.0)	
N+, *n* (%)	20 (45.5)	22 (56.4)	13 (52.0)	0.604
M+, *n* (%)	1 (2.3)	2 (5.6)	2 (8.0)	0.619
G3 histology, *n* (%)	25 (56.8)	27 (69.2)	21 (87.5)	0.034*
ER+, *n* (%)	2 (4.6)	4 (10.3)	3 (12.0)	0.243
Low	30 (68.2)	19 (48.7)	11 (44.0)	
Positive				
PgR+, *n* (%)	29 (65.9)	19 (48.7)	11 (44.0)	0.139
Ki67-high, *n* (%)	29 (65.9)	30 (76.9)	21 (84.0)	0.226
HER2+, *n* (%)				0.468
Low	14 (31.8)	9 (23.0)	6 (24.0)	
Positive	3 (6.8)	7 (18.0)	4 (16.0)	
Subtypes, *n* (%)				0.385
HR+/HER2–	28 (63.6)	19 (48.7)	11 (44.0)	
HER2+	3 (6.8)	7 (18.0)	4 (16.0)	
HR-/HER2–	13 (29.6)	13 (33.3)	10 (40.0)	

SD, standard deviation; NST, no special type; LVI, lymph vascular invasion; ER, estrogen receptor; PgR, progesterone receptor. Significant associations (p < 0.05) are highlighted with an asterisk (*).

### 3.2 Gestational age-dependent variations in the tumor microenvironment of PrBC

**Figure 2:**
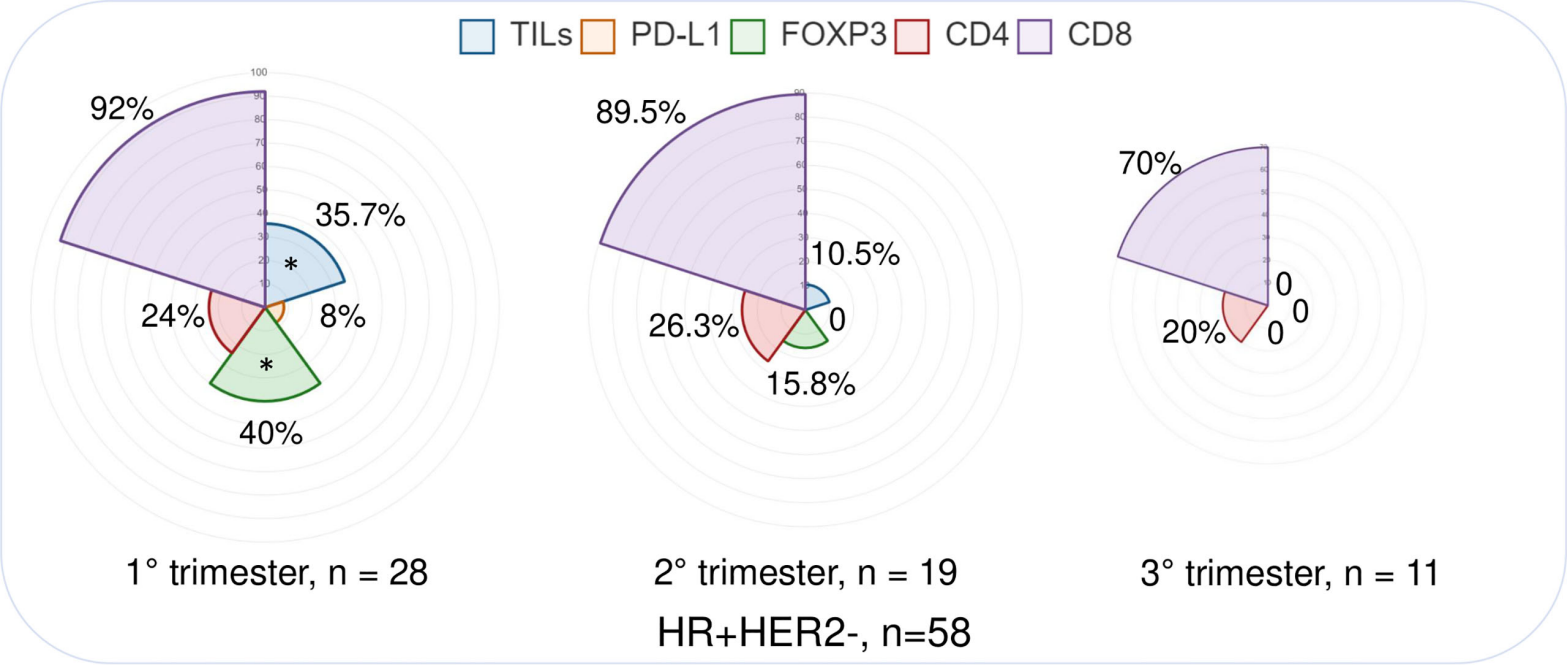
Immunograms showing the distribution of selected immune-related features in different pregnancy trimesters, focusing on the HR+/HER2− subtype. PrBC, breast cancer during pregnancy; HRs, hormone receptors; TNBC, triple-negative breast cancer; PD-L1, programmed death-ligand 1; TILs, tumor-infiltrating lymphocytes; FOXP3, forkhead box P3. Significant correlations among the different subset of patients (color-coded based on the legends) are highlighted with a star (*).

The analysis of TME dynamics across trimesters in the overall PrBC population did not reveal any statistically significant differences (**Supplementary Table S3**). However, when examining the tumor subtypes, we observed distinct patterns in HR+/HER2– breast cancers. In this subgroup of PrBC, the proportion of tumors with high/moderate TILs was significantly higher in the early phases of pregnancy compared to the later phases (first trimester: *n* = 10, 35.7%; second trimester: *n* = 2, 10.5%; third trimester: *n* = 0; *p* = 0.02). This finding corresponded to a higher proportion of patients with FOXP3+ TILs in the first months, which progressively decreased (first trimester: *n* = 10, 40%; second trimester: *n* = 3, 15.8%; third trimester: *n* = 0; *p* = 0.03), as shown in **Table 2** and **Figure 2**. These findings highlight the dynamic changes in the TME of PrBC, specifically in HR+/HER2– tumors, with variations in immune composition based on gestational age.

**Table 2:** Distribution of tumor-infiltrating lymphocyte (TIL) subpopulations and PD-L1 expression across pregnancy trimesters, categorized by breast cancer subtypes.

	HR+/HER2− *n* = 58				HR-/HER2− *n* = 36				HER2+ *n* = 14			
	First trimester *n* = 28	Second trimester *n* = 19	Third trimester *n* = 11	*p*-value	First trimester *n* = 13	Second trimester *n* = 13	Third trimester *n* = 10	*p*-value	First trimester *n* = 3	Second trimester *n* = 7	Third trimester *n* = 4	*p*-value
TILs, *n* (%)				0.022*				0.245				0.539
≤20%	18 (64.3)	17 (89.5)	11 (100.0)		11 (84.6)	8 (61.5)	5 (50.0)		2 (66.7)	5 (71.4)	4 (100.0)	
>20%	10 (35.7)	2 (10.5)	0 (0.0)		2 (15.4)	5 (38.5)	5 (50.0)		1 (33.3)	2 (28.6)	0 (0.0)	
PD-L1 (CPS), *n* (%)				0.668				0.068				–
<10	23 (92.0)	19 (100.0)	10 (100.0)		11 (100.0)	13 (100.0)	7 (77.8)		2 (100.0)	7 (100.0)	3 (100.0)	
≥10	2 (8.0)	0 (0.0)	0 (0.0)		0 (0.0)	0 (0.0)	2 (22.2)		0 (0.0)	0 (0.0)	0 (0.0)	
PD-L1 (CPS), *n* (%)				0.668				0.068				–
<10	23 (92.0)	19 (100.0)	10 (100.0)		11 (100.0)	13 (100.0)	7 (77.8)		2 (100.0)	7 (100.0)	3 (100.0)	
≥10	2 (8.0)	0 (0.0)	0 (0.0)		0 (0.0)	0 (0.0)	2 (22.2)		0 (0.0)	0 (0.0)	0 (0.0)	
FOXP3, *n* (%)				0.028*				1.000				0.276
<1%	15 (60.0)	16 (84.2)	10 (100.0)		6 (54.6)	6 (46.2)	5 (55.6)		1 (50.0)	4 (57.1)	4 (100.0)	
≥1%	10 (40.0)	3 (15.8)	0 (0.0)		5 (45.4)	7 (53.8)	4 (44.4)		1 (50.0)	3 (42.9)	0 (0.0)	
CD4, *n* (%)				1.000				0.194				0.746
<1%	19 (76.0)	14 (73.7)	8 (80.0)		9 (81.8)	10 (76.9)	4 (44.4)		2 (100.0)	4 (57.1)	2 (66.7)	
≥1%	6 (24.0)	5 (26.3)	2 (20.0)		2 (18.2)	3 (23.1)	5 (55.6)		0 (0.0)	3 (42.9)	1 (33.3)	
CD8, *n* (%)				0.224				0.075				1.000
<1%	2 (8.0)	2 (10.5)	3 (30.0)		5 (45.5)	1 (7.7)	7 (11.1)		0 (0.0)	2 (28.6)	0 (0.0)	
≥1%	23 (92.0)	2 (10.5)	7 (70.0)		6 (55.5)	12 (92.3)	8 (88.9)		2 (100.0)	5 (71.4)	3 (100.0)	

HR, hormone receptors; FOXP3, forkhead box P3; PD-L1, programmed death-ligand 1; CPS, combined positive score. Significant associations (p < 0.05) are highlighted with an asterisk (*).

### 3.3 Loss of PD-L1 expression as a potential indicator of disease recurrence in early pregnancy

The incidence of disease recurrence and death did not show significant differences among the trimesters, as detailed in **Supplementary Figure S1**, **Supplementary Table S4**, and **Supplementary Table S5**. Additionally, a higher proportion of patients who experienced disease recurrence or death showed a lack or low presence of TILs, as well as the absence of FOXP3+ and CD4+ cells. In contrast, the presence of CD8+ TILs was predominantly observed in patients with worse clinical outcomes, although statistical significance was not reached. These observations were confirmed after stratification for PD-L1 status. However, when considering score = 1 as a cutoff value for CPS, a higher proportion of patients with PD-L1 negative tumors experienced disease recurrence compared to those with CPS ≥ 1. This trend was observed across all trimesters, but statistical significance was limited to the first trimester in both endocrine therapy and chemotherapy groups (*n* = 9, 100% vs. *n* = 9, 56.3%; *p* = 0.03 and *n* = 9, 100% vs. *n* = 7, 53.9%; *p* = 0.02, respectively), as shown in **Tables 3**, **4**. Similarly, a higher frequency of deceased patients had a lack of PD-L1 expression compared to those who survived during the follow-up period, although statistical significance was not reached (as indicated in **Supplementary Table S6** and **Supplementary Table S7**). These results suggest that the progression of PrBC may be influenced by PD-L1 expression, observed during the early stages of pregnancy.

**Table 3:** Disease progression based on the tumor immune characteristics in patients treated with endocrine therapy.

	Disease recurrence after endocrine treatment (*n* = 64)									
	First trimester			Second trimester				Third trimester		
	No (*n* = 19)	Yes (*n* = 9)	*p*-value	No (*n* = 13)	Yes (*n* = 10)	*p*-value		No (*n* = 9)	Yes (*n* = 4)	*p*-value
TILs, *n* (%)			1.000			1.000				0.308
≤20%	12 (63.2)	6 (66.7)		12 (92.3)	9 (90.0)			9 (100.0)	3 (75.0)	
>20%	7 (36.8)	3 (33.3)		1 (7.7)	1 (10.0)			0 (0.0)	1 (25.0)	
PD-L1(CPS), *n* (%)			0.027*			1.000				1.000
<1	9 (56.3)	9 (100.0)		11 (84.6)	9 (90.0)			7 (87.5)	4 (100.0)	
≥1	7 (43.8)	0 (0.0)		2 (15.4)	1 (10.0)			1 (12.5)	0 (0.0)	
FOXP3, *n* (%)			1.000			1.000				0.333
<1%	8 (50.0)	5 (55.6)		11 (84.6)	8 (80.0)			8 (100.0)	3 (75.0)	
≥1%	8 (50.0)	4 (44.4)		2 (15.4)	2 (20.0)			0 (0.0)	1 (25.0)	
CD4, *n* (%)			0.364			0.339				0.548
<1%	11 (68.8)	8 (88.9)		9 (69.2)	9 (90.0)			6 (75.0)	2 (50.0)	
≥1%	5 (31.3)	1 (11.1)		4 (30.8)	1 (10.0)			2 (25.0)	2 (50.0)	
CD8, *n* (%)			1.000			0.281				0.491
<1%	2 (12.5)	1 (11.1)		1 (7.7)	3 (30.0)			3 (37.5)	0 (0.0)	
≥1%	14 (87.5)	8 (88.9)		12 (92.3)	7 (70.0)			5 (62.5)	4 (100.0)	

TILs, tumor-infiltrating lymphocytes; FOXP3, forkhead box P3; PD-L1, programmed death-ligand 1; CPS, combined positive score. Significant associations (p < 0.05) are highlighted with an asterisk (*).

**Table 4:** Disease progression based on the tumor immune characteristics in patients treated with chemotherapy.

	Disease recurrence after chemotherapy (*n* = 82)									
	First trimester			Second trimester				Third trimester		
	No (*n* = 16)	Yes (*n* = 12)	*p*-value	No (*n* = 17)	Yes (*n* = 15)	*p*-value		No (*n* = 13)	Yes (*n* = 9)	*p*-value
TILs, *n* (%)			1.000			0.229				1.000
≤20%	11 (68.8)	9 (75.0)		11 (64.7)	13 (86.7)			10 (76.9)	7 (77.8)	
>20%	5 (31.2)	3 (25.0)		6 (35.3)	2 (13.3)			3 (23.1)	2 (22.2)	
PD-L1(CPS), *n* (%)			0.019*			1.000				1.000
<1	7 (53.9)	10 (100.0)		12 (70.6)	11 (73.3)			7 (70.0)	7 (77.8)	
≥1	6 (46.2)	0 (0.0)		5 (29.4)	4 (26.7)			3 (30.0)	2 (22.2)	
FOXP3, *n* (%)			0.680			0.131				0.285
<1%	6 (46.2)	6 (60.0)		8 (47.1)	11 (73.3)			10 (90.9)	6 (66.7)	
≥1%	7 (53.8)	4 (40.0)		9 (52.9)	4 (26.7)			1 (9.1)	3 (33.3)	
CD4, *n* (%)			0.089			0.389				1.000
<1%	7 (53.9)	9 (90.0)		10 (58.8)	11 (73.3)			6 (60.0)	5 (55.6)	
≥1%	7 (53.9)	9 (90.0)		10 (58.8)	11 (73.3)			6 (60.0)	5 (55.6)	
CD8, *n* (%)			0.604			0.319				1.000
<1%	3 (23.1)	1 (10.0)		1 (5.9)	3 (20.0)			2 (20.0)	1 (11.1)	
≥1%	10 (76.9)	9 (90.0)		1 (5.9)	3 (20.0)			2 (20.0)	1 (11.1)	

TILs, tumor-infiltrating lymphocytes; FOXP3, forkhead box P3; PD-L1, programmed death-ligand 1; CPS, combined positive score. Significant associations (p < 0.05) are highlighted with an asterisk (*).

## 4 Discussion

In this study, we characterized the PrBC immune landscape dynamics based on gestational age and demonstrated that the anti-tumor immune response varies throughout pregnancy. Our study unveiled diverse immunological patterns across trimesters, linked to distinct clinical outcomes. With increasing gestational age, tumor behavior became more aggressive. TIL composition varied notably throughout trimesters, with a higher proportion of tumors having high/moderate TILs and FOXP3+ cells in early pregnancy, gradually declining over time. Notably, low PD-L1 expression was associated with first-trimester disease relapse.

Certain clinicopathologic characteristics in PrBC, such as advanced stages at diagnosis, high grade, and increased lymph node involvement, can vary throughout each trimester of pregnancy (42–45). It has been previously observed that breast tumors in the later stages of pregnancy are significantly more frequently of a higher grade compared to those in the first trimester (3, 6, 25). Consistent with this, our findings showed an increased prevalence of poorly differentiated neoplasms with advancing gestational age. Not surprisingly, a lower proportion of patients had stage I tumors in later pregnancy phases, confirming the relationship between advanced gestational age and breast cancer aggressiveness. We also observed a non-significant but more pronounced proportion of Ki67 high score, LVI, and nodal involvement in the third trimester. Taken together, these findings suggest more aggressive tumor biology and provide the potential rationale for adjusting the management of high-risk individuals.

When considering the fetus as a graft, it is intriguing to contemplate the deliberate and regulated response of the maternal immune system, which has implications for both the TME and the host’s overall immune capabilities. Evaluating different breast cancer subtypes, we found that TILs were higher in early pregnancy but decreased as gestation progressed in HR+/HER2– PrBC, suggesting a progressive increase in tumor immune tolerance. High TILs in breast cancer are linked to better long-term outcomes (46–48). Consistent with this, our findings revealed that patients with worse clinical behavior were more common in the last trimester, where tumors with high/moderate TILs were less frequently observed. FOXP3+ TILs were higher in early pregnancy, gradually decreasing, confirming their role in establishing immune tolerance (16, 49). Analyzing patient survival, we discovered that all cases of disease recurrence in the first trimester were PD-L1 negative, irrespective of therapy, indicating the impact of TME dynamics, like PD-L1 expression, in early pregnancy on PrBC outcomes. These results suggest that the immune response within HR+/HER2– breast cancers varies throughout pregnancy, with a higher presence of TILs and FOXP3+ TILs in the early stages. This may indicate a more active immune response against the tumor during the initial months, potentially contributing to better clinical outcomes. As the pregnancy progresses, the proportion of tumors with high/moderate TILs and FOXP3+ TILs decrease, suggesting a potential shift in the immune landscape and immune tolerance mechanisms. During pregnancy, the maternal host undergoes adaptive changes in the immune system to protect the semi-allogenic fetoplacental unit, involving attenuation of adaptive immunity and protection from innate immune defense mechanisms (50). Malignant cells can modify metabolism and signaling pathways in the TME to enhance their survival (51). These modifications can occur through various mechanisms, including the regulation of Tregs (52). Tregs play a role in immunological tolerance and can contribute to tumor immune evasion by suppressing immune responses against cancer cells. By modulating the TME and influencing the activity of Tregs, malignant cells can create an environment that supports their survival and growth. Treg cells increase during early pregnancy, likely due to their role in implantation and placental invasion of maternal tissues (49, 53, 54). Upregulated PD-L1 expression in breast cancer contributes to immunosuppression by binding to PD-1 and suppressing T-cell response (55–57). Our findings highlight the importance of tailored clinical management based on trimester and immunological profile in PrBC.

This study has limitations that should be acknowledged. Firstly, using TMAs to assess biomarker expression may not fully capture intratumor heterogeneity. To address this, we performed re-analysis on corresponding full-face sections when heterogeneity was observed. Additionally, the small sample size and potential confounding factors may impact the clinical significance of our results, particularly for HER2+ and TNBC. Further multicentric studies are needed to gain a comprehensive understanding in these subgroups. The use of a limited IHC panel with only four immune biomarkers is another inherent limitation. Expanding the examination with spatial and multiplex technologies would provide deeper insights into the immune dynamics in PrBC. Furthermore, owing to the retrospective nature of the study, comprehensive data on specific lifestyle factors were not available. Future studies should consider incorporating detailed information on lifestyle factors to enhance our understanding of PrBC. Despite these limitations, our findings offer novel insights into the TME and biology of PrBC, potentially linking to the clinical course of patients.

In conclusion, our study suggests that immune tolerance events are involved in early gestational PrBC and that decreased TILs and FOXP3 in later months may contribute to disease aggressiveness. Understanding similarities and differences between the maternal immune system and the TME provides novel insights for tailored patient management. Consideration of trimester-specific immune profiles is important for PrBC clinical decision-making. Further research is needed to uncover underlying mechanisms and their impact on outcomes.

## Data availability statement

The original contributions presented in the study are included in the article/**Supplementary Material**. Further inquiries can be directed to the corresponding author.

## Ethics statement

The study was approved by the local Ethical Committees under protocol numbers #620_2018bis and #UID3472. The patients/participants provided their written informed consent to participate in this study.

## Author contributions

Conceptualization, FP, NF, and EG-R. Methodology, ES and NF. Histological assessment, NF. Statistical analysis, ES and MN. Resources, SF, GV, and EG-R. Data curation, ES. Writing—original draft preparation, ES, KV, and MI. Review, CB, EL, GS, SM, PV, GV, VG, FP, and EG-R. Writing and editing, ES and NF. Figures, NF and ES. Supervision, NF. All authors contributed to the article and approved the submitted version.
