# Supplementary material for: Immune microenvironment dynamics in breast cancer during pregnancy: impact of gestational age on tumor-infiltrating lymphocytes and prognosis
Source: Front Oncol. 2023 Aug 21;13:1116569. doi: 10.3389/fonc.2023.1116569 (PMC10475935; doi:10.3389/fonc.2023.1116569)
Supplement: Supplementary file 1 [file DataSheet_1.docx]

Supplementary Material

Immune microenvironment dynamics in breast cancer during pregnancy: Impact of gestational age on tumor-infiltrating lymphocytes and prognosis

Elham Sajjadi ^1,2‡^, Konstantinos Venetis ^1,2‡^, Mariia Ivanova ^2^, Marianna Noale ^3^, Concetta Blundo ^4^, Eugenia Di Loreto ^5^, Giovanna Scarfone ^5^, Stefano Ferrero ^6,7^, Stefania Maggi ^3^, Paolo Veronesi ^1,8^, Viviana E. Galimberti ^8^, Giuseppe Viale ^1,2^, Nicola Fusco^1,2, +^*, Fedro A. Peccatori ^9, +^, Elena Guerini-Rocco^1,2, +^

^1^ Department of Oncology and Hemato-Oncology, University of Milan, Via Festa del Perdono 7, 20122 Milan, Italy;

^2^ Division of Pathology, IEO, European Institute of Oncology IRCCS, Via Giuseppe Ripamonti 435, 20141 Milan, Italy;

^3^ National Research Council (CNR), Neuroscience Institute Aging Branch, Via Giustiniani 2, 35128 Padua, Italy;

^4^ Breast Surgery Unit, Fondazione IRCCS Ca’ Granda—Ospedale Maggiore Policlinico, 20122 Milan, Italy;

^5^ Gynecology Unit, Fondazione IRCCS Ca’ Granda—Ospedale Maggiore Policlinico, 20122 Milan, Italy;

^6^ Division of Pathology, Fondazione IRCCS Ca’ Granda—Ospedale Maggiore Policlinico, 20122 Milan, Italy;

^7^ Department of Biomedical, Surgical, and Dental Sciences, University of Milan, Via Festa del Perdono 7, 20122 Milan, Italy;

^8^ Division of Breast Surgery, IEO, European Institute of Oncology IRCCS, 20141 Milan, Italy;

^9^ Fertility and Procreation Unit, Division of Gynecologic Oncology, IEO European Institute of Oncology IRCCS, 20141 Milan, Italy.

^‡^ These authors contributed equally to this work

^+^ These authors jointly directed this work.

*** Correspondence:**[nicola.fusco@ieo.it](mailto:nicola.fusco@ieo.it); +39-02-9437-2079

# Supplementary Figure and Tables


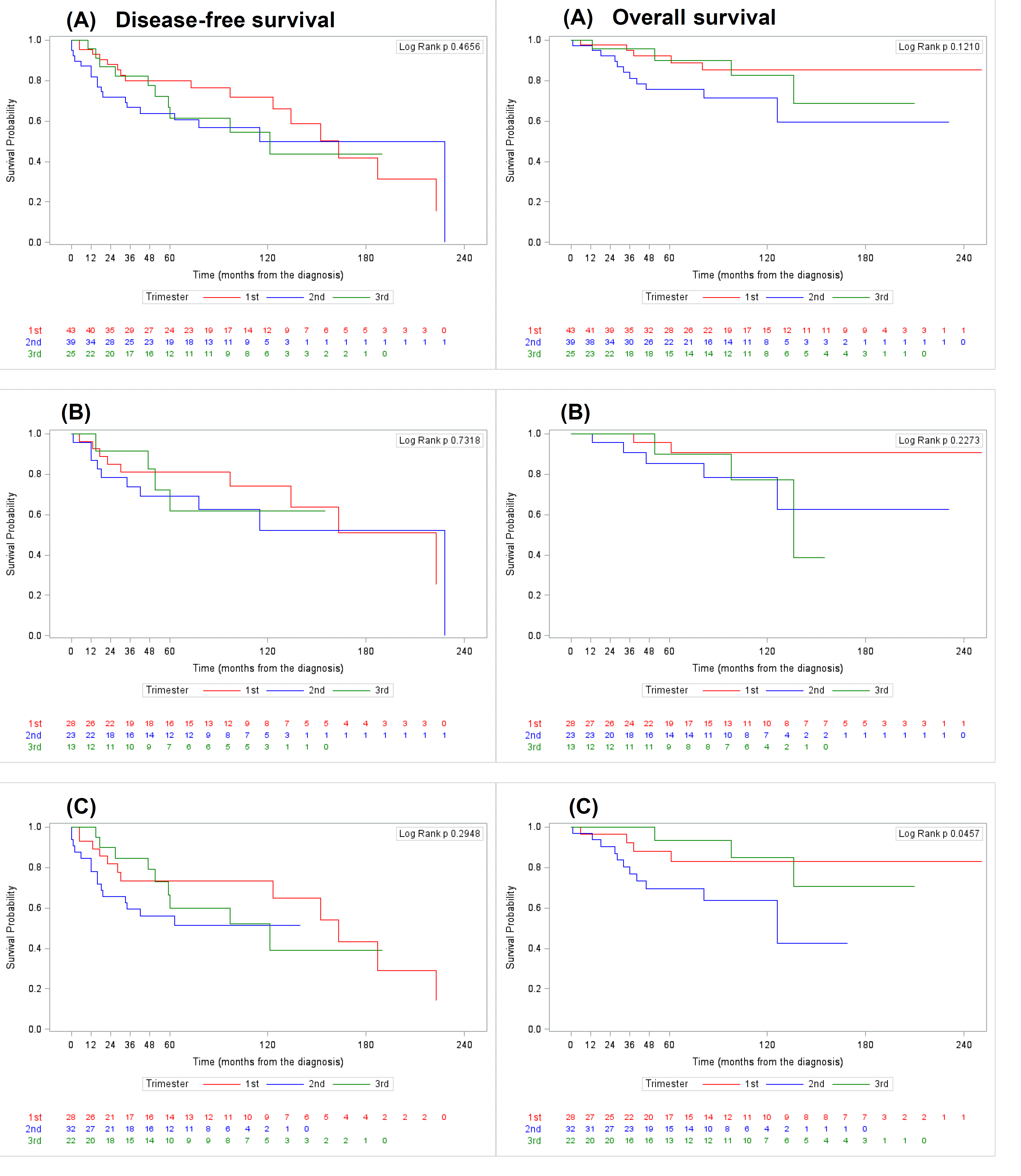


**Supplementary Figure 1.** Kaplan-Meier curves describing the disease-free survival and overall survival analysis of PrBC patients according to the trimester of pregnancy. (A) Overall population; (B) Endocrine therapy treated population; (C) Chemotherapy treated population.

| **Marker** | **Clone** | **Dilution** | **Antigen retrieval** | **Scoring** |
| --- | --- | --- | --- | --- |
| ER | EP1 | RTU | High pH, 20' | ASCO/CAP and St Gallen guidelines; positive if ≥1% of tumor cell nuclei are immunoreactive. |
| PgR | PgR 636 | 1:100 | High pH, 30’ | ASCO/CAP and St Gallen guidelines; positive if ≥1% of tumor cell nuclei are immunoreactive. |
| Ki-67 | MIB1 | RTU | High pH, 30’ | International Ki67 in Breast Cancer Working Group; high if ≥30% of tumor cell nuclei are immunoreactive |
| HER2 | Polyclonal | 1:400 | Low pH, 30' | ASCO/CAP guidelines; 3+ if complete membrane staining that is intense and >10% of tumor cells; 2+ if weak to moderate complete membrane staining in >10% of tumor cells or complete membrane staining that is intense but within ≤10% of tumor cells; 1+ if incomplete membrane staining that is faint/barely perceptible and within >10% of tumor cells; 0 if no staining observed or membrane stating that is incomplete and is faint/barely perceptible and within ≤10% of tumor cells. |
| TILs | n/a | n/a | n/a |  |
| CD4 | 4B12 | RTU | Low pH, 30' | Negative if complete loss of membrane staining within the lymphocytes. |
| CD8 | C8/144B | 1:50 | Low pH, 30' | Negative if complete loss of membrane staining within the lymphocytes; low, 1-30%; intermediate, 31-50%; high, >50%. |
| FOXP3 | Polyclonal | 1:200 | Low pH, 30' | Negative if complete loss of membrane staining within the lymphocytes. |
| PD-L1 | 22C3 | RTU | Low pH, 30' | Combined positive score (CPS), i.e. total number of PD-L1+ tumor cells, lymphocytes, and macrophages divided by the total number of viable tumor cells, multiplied by 100. |
| **Supplementary Table S1**. List of antibodies used for immunohistochemical analyses. The analyses were performed on a Dako Omnis staining platform using the polymer-based EnVision FLEX antigen retrieval system. PD-L1, programmed death-ligand 1; RTU, ready to use. | | | | |

|  | **3^rd^ trimester, n=25** | | | |
| --- | --- | --- | --- | --- |
|  | **HR+/HER2-**  **n=11** | **HER2+**  **n=4** | **HR-/HER2-n=10** | **p-value** |
| High Grade, n (%) | 8 (81.8) | 4 (100) | 9 (90) | 0.355 |
| **Supplementary Table S2.** Correlation between high histologic grade (G3) with breast cancer subtypes in the patients who were diagnosed with PrBC in the last trimester. PrBC, breast cancer during pregnancy; HR, hormone receptors. Significant associations (p<0.05) are highlighted with a star (*). | | | | |

|  | **Total population**  **n=108** | | | |
| --- | --- | --- | --- | --- |
|  | **1^st^ trimester**  **n=44** | **2^nd^ trimester**  **n=39** | **3rd trimester**  **n=25** | **p-value** |
| TILs, n (%)  Zero or Low  Intermediate or High | 31 (70.5)  13 (29.5) | 30 (76.9)  9 (23.1) | 20 (80.0)  5 (20.0) | 0.639 |
| PD-L1CPS, n (%)  <10  ≥10 | 36 (94.7)  2 (5.3) | 39 (100.0)  0 (0.0) | 20 (90.9)  2 (9.1) | 0.118 |
| FOXP3, n (%)  Absence  Presence | 22 (57.9)  16 (42.1) | 26 (66.7)  13 (33.3) | 19 (82.6)  4 (17.4) | 0.138 |
| CD4, n (%)  Absence  Presence | 30 (79.0)  8 (21.0) | 28 (71.8)  11 (28.2) | 14 (63.6)  8 (36.4) | 0.433 |
| CD8, n (%)  Absence  Presence | 7 (18.4)  31 (81.6) | 5 (12.8)  34 (87.2) | 4 (18.2)  18 (81.8) | 0.767 |
| **Supplementary Table S3.** Relative prevalence of TILs subpopulation in the tumor stroma and PD-L1 expression among trimesters. PrBC, breast cancer during pregnancy; TILs, tumor-infiltrating lymphocytes; FOXP3, forkhead box P3; PD-L1, programmed death-ligand 1; CPS, combined positive score. Significant associations (p<0.05) are highlighted with a star (*). | | | | |

|  | **All the patients**  **(n=107)** | | | **Chemotherapy+**  **(n=82)** | | | **Hormone therapy+**  **(n=64)** | | |
| --- | --- | --- | --- | --- | --- | --- | --- | --- | --- |
|  | **Disease recurrence** | | | **Disease recurrence** | | | **Disease recurrence** | | |
|  | No  (n=63) | Yes  (n=44) | p-value | No  (n=46) | Yes  (n=36) | p-value | No  (n=41) | Yes  (n=23) | p-value |
| 1^st^ trimester | 27 (62.8) | 16 (37.2) | 0.707 | 16 (57.1) | 12 (42.9) | 0.902 | 19 (67.9) | 9 (32.1) | 0.640 |
| 2^nd^ trimester | 21 (53.9) | 18 (46.2) |  | 17 (53.1) | 15 (46.9( |  | 13 (56.5) | 10 (43.5) |  |
| 3^rd^ trimester | 15 (60.0) | 10 (40.0) |  | 13 (59.1) | 9 (40.9) |  | 9 (69.2) | 4 (30.8) |  |
| **Supplementary Table S4.** Disease progression status based on the trimesters in different therapeutic approaches. Significant associations (p<0.05) are highlighted with a star (*). | | | | | | | | | |

|  | **All the patients**  **(n=107)** | | | **Chemotherapy+**  **(n=82)** | | | **Hormone therapy+**  **(n=64)** | | |
| --- | --- | --- | --- | --- | --- | --- | --- | --- | --- |
|  | **Death** | | | **Death** | | | **Death** | | |
|  | No  (n=87) | Yes  (n=20) | p-value | No  (n=64) | Yes  (n=18) | p-value | No  (n=54) | Yes  (n=10) | p-value |
| 1^st^ trimester | 38 (88.4) | 5 (11.6) | 0.146 | 24 (85.7) | 4 (14.3) | 0.094 | 26 (92.9) | 2 (7.1) | 0.244 |
| 2^nd^ trimester | 28 (71.8) | 11 (28.2) |  | 21 (65.6) | 11 (34.4) |  | 18 (78.3) | 5 (21.7) |  |
| 3^rd^ trimester | 21 (84.0) | 4 (16.0) |  | 19 (86.4) | 3 (13.6) |  | 10 (76.9) | 3 (23.1) |  |
| **Supplementary Table S5.** Death status based on the trimesters in different therapeutic approaches. Significant associations (p<0.05) are highlighted with a star (*). | | | | | | | | | |

|  | **Death (in Hormone therapy+: n=64)** | | | | | | | | |
| --- | --- | --- | --- | --- | --- | --- | --- | --- | --- |
|  | **1st trimester** | | | **2nd trimester** | | | **3rd trimester** | | |
|  | **No**  **(n=26)** | **Yes**  **(n=2)** | **p-**  **value** | **No**  **(n=18)** | **Yes**  **(n=5)** | **p-**  **value** | **No**  **(n=10)** | **Yes**  **(n=3)** | **p-**  **value** |
| TILs, n (%)  0 or Low  Intermediate or High | 17 (65.4)  9 (34.6) | 1 (50.0)  1 (50.0) | 1.000 | 16 (88.9)  2 (11.1) | 5 (100.0)  0 (0.0) | 1.000 | 10 (100.0)  0 (0.0) | 2 (66.7)  1 (33.3) | 0.231 |
| PD-L1CPS, n (%)  0  ≥1 | 16 (69.6)  7 (30.4) | 2 (100)  0 (0.0) | 1.000 | 16 (88.9)  2 (11.1) | 4 (80.0)  1 (20.0) | 0.539 | 8 (88.9)  1 (11.1) | 2 (100.0)  0 (0.0) | 1.000 |
| FOXP3, n (%)  Absence  Presence | 12 (52.2)  11 (47.8) | 1 (50.0)  1 (50.0) | 1.000 | 15 (83.3)  3 (16.7) | 4 (80.0)  1 (20.0) | 1.000 | 9 (100.0)  0 (0.0) | 2 (66.7)  1 (33.3) | 0.250 |
| CD4, n (%)  Absence  Presence | 17 (73.9)  6 (26.1) | 2 (100)  0 (0.0) | 1.000 | 13 (72.2)  5 (27.8) | 5 (100)  0 (0.0) | 0.545 | 6 (66.7)  3 (33.3) | 2 (66.7)  1 (33.3) | 1.000 |
| CD8, n (%)  Absence  Presence | 3 (13.0)  20 (87.0) | 0 (0.0)  2 (100) | 1.000 | 2 (11.1)  16 (88.9) | 2 (40.0)  3 (60.0) | 0.194 | 3 (33.3)  6 (66.7) | 0 (0.0)  3 (100.0) | 0.509 |
| **Supplementary Table S6.** Death occurrence according to TILs populations status in patients with a history of hormone therapy. treatment. TILs, tumor-infiltrating lymphocytes; FOXP3, forkhead box P3; PD-L1, programmed death-ligand 1; CPS, combined positive score. Significant associations (p<0.05) are highlighted with a star (*). | | | | | | | | | |

|  | **Death (in chemotherapy+: n=82)** | | | | | | | | |  |
| --- | --- | --- | --- | --- | --- | --- | --- | --- | --- | --- |
|  | **1^st^ trimester** | | | **2^nd^ trimester** | | | **3^rd^ trimester** | | |  |
|  | **No**  **(n=24)** | **Yes**  **(n=4)** | **p-**  **value** | **No**  **(n=21)** | **Yes**  **(n=11)** | **p-**  **value** | **No**  **(n=19)** | **Yes**  **(n=3)** | **p-**  **value** | |
| TILs, n (%)  0 or Low  Intermediate or High | 17 (70.8)  7 (29.2) | 3 (75.0)  1 (25.0) | 1.000 | 14 (66.7)  7 (33.3) | 10 (90.9)  1 (9.1) | 0.209 | 15 (79.0)  4 (21.0) | 2 (66.7)  1 (33.3) | 1.000 | |
| PD-L1CPS, n (%)  0  ≥1 | 14 (70.0)  6 (30.0) | 3 (100.0)  0 (0.0) | 0.539 | 16 (76.2)  5 (23.8) | 7 (63.6)  4 (36.4) | 0.681 | 11 (68.8)  5 (31.3) | 3 (100.0)  0 (0.0) | 0.530 | |
| FOXP3, n (%)  Absence  Presence | 10 (50.0)  10 (50.0) | 2 (66.7)  1 (33.3) | 1.000 | 10 (47.6)  11 (52.4) | 9 (81.8)  2 (18.2) | 0.128 | 14 (82.4)  3 (17.7) | 2 (66.7)  1 (33.3) | 0.509 | |
| CD4, n (%)  Absence  Presence | 13 (65.0)  7 (35.0) | 3 (100.0)  0 (0.0) | 0.526 | 11 (52.4)  10 (47.6) | 10 (90.9)  1 (9.1) | 0.050 | 9 (56.3)  7 (43.8) | 2 (66.7)  1 (33.3) | 1.000 | |
| CD8, n (%)  Absence  Presence | 4 (20.0)  16 (80.0) | 0 (0.0)  3 (100.0) | 1.000 | 1 (4.8)  20 (95.2) | 3 (27.3)  8 (72.7) | 0.106 | 3 (18.8)  13 (81.3) | 0 (0.0)  3 (100.0) | 1.000 | |
| **Supplementary Table S7.** Death occurrence according to TILs populations status in patients with a history of chemotherapy. treatment. TILs, tumor-infiltrating lymphocytes; FOXP3, forkhead box P3; PD-L1, programmed death-ligand 1; CPS, combined positive score. Significant associations (p<0.05) are highlighted with a star (*). | | | | | | | | | | |
